# Late and Severe Myopathy in a Patient With Glycogenosis VII Worsened by Cyclosporine and Amiodarone

**DOI:** 10.3389/fneur.2019.00077

**Published:** 2019-02-07

**Authors:** Massimiliano Filosto, Stefano Cotti Piccinelli, Anna Pichiecchio, Olimpia Musumeci, Anna Galvagni, Filomena Caria, Serena Gallo Cassarino, Enrico Baldelli, Raimondo Vitale, Alessandro Padovani, Antonio Toscano

**Affiliations:** ^1^Unit of Neurology, Center for Neuromuscular Diseases, ASST “Spedali Civili” and University of Brescia, Brescia, Italy; ^2^IRCCS Mondino Foundation, Pavia, Italy; ^3^Department of Brain and Behavioural Sciences, University of Pavia, Pavia, Italy; ^4^Department of Clinical and Experimental Medicine, UOC di Neurologia e Malattie Neuromuscolari, University of Messina, Messina, Italy

**Keywords:** glycogenosis VII, muscle MRI, PFK deficiency, cyclosporine, amiodarone, GSD VII

## Abstract

Glycogenosis VII (GSD VII) is a rare autosomal recessive glycogen storage disorder caused by mutations in the *PFKM* gene encoding the phosphofructokinase (PFK) enzyme. A classical form with exercise intolerance, contractures, and myoglobinuria, a severe multisystem infantile form, an hemolytic variant and a late-onset form usually presenting with muscle pain and mild fixed proximal weakness have been reported. We describe a 65-year-old man affected by muscle PFK deficiency who, since the age of 33, presented with exercise intolerance and myoglobinuria. Muscle biopsy showed a vacuolar myopathy with glycogen storage. The biochemical assay of PFK-M showed very low residual activity (6%). Genetic analysis of *PFKM* gene evidenced the presence of the heterozygote c.1817A>C (p.Asp543Ala) and c.488 G>A (p.Arg100Gln) pathogenic mutations. In his fifth decade, he started cyclosporine after liver transplantation for hepatocellular carcinoma and, then, amiodarone because of atrial fibrillation. In the following years, he developed a progressive and severe muscle weakness, mainly involving lower limbs, up to a loss of independent walking. Muscle MRI showed adipose substitution of both anterior and posterior thigh muscles with selective sparing of the medial compartment. Marked signs of adipose substitution were also documented in the legs with a selective replacement of gemelli and peroneus muscles. The temporal relationship between the patient's clinical worsening and chronic treatment with cyclosporine and amiodarone suggests an additive toxic damage by these two potentially myotoxic drugs determining such an unusually severe phenotype, also confirmed by muscle MRI findings.

## Background

Glycogenosis VII (GSD VII; Tarui's Disease) is a rare autosomal recessive glycogen storage disorder caused by mutations in the *PFKM* gene encoding the muscle phosphofructokinase (PFK) enzyme (1).

PFK catalyzes the phosphorylation of fructose-6-phosphate to fructose-1,6-bisphosphate, which is a rate-limiting step in the glycolytic pathway (2).

PFK is a tetrameric enzyme consisting of three different subunits: PFK-L (liver), PFK-M (muscle), and PFK-P (platelet). The combination of these subunits varies in different tissues.

In GSD VII, inhibition of the PFK-M function prevents the formation of adenosine triphosphate which results in a lack of energy for muscles during strenuous exercise (1, 3).

GSD VII presents with 4 different clinical forms: (a) a severe and rapidly progressive infantile form, (b) the haemolytic form with no muscle involvement, (c) the classical GSD VII phenotype including childhood onset of exercise intolerance and myoglobinuria, and (d) the late-onset form characterized by mild fixed proximal weakness (1, 3, 4).

On the other hand, it is known that several commonly prescribed drugs may cause adverse effects on muscle tissue, that more frequently occur in patients with a preexisting myopathy.

Among these drugs, cyclosporine is an immunophilin which is used as immunosuppressive agent in patients receiving organ transplantation (5). Amiodarone is an antiarrhythmic amphiphilic drug containing hydrophilic and hydrophobic domains which interacts with phospholipids of cell membranes and organelles (5). Both drugs are known to cause, although quite rarely, acute myotoxicity, often triggered by the contemporary administration of statins (5).

We describe herein the unusual clinical course of a patient with GSD VII which, since the fifth decade, after starting long-term treatment of cyclosporine and, later, amiodarone, developed a progressive severe worsening of muscle weakness with prevailing involvement of lower limbs.

## Case Presentation

This patient has been originally reported in Tsujino et al. (6). At the time of the first diagnosis, he was a 43-year-old man who, since childhood, could not keep up with his peers in physical activities. Since the age of 33, he complained of a mild proximal muscle weakness, mainly in the lower limbs, exercise intolerance, hyperCKemia, and myoglobinuria.

Muscle biopsy showed a vacuolar myopathy with glycogen storage. Biochemical assay of PFK-M showed a residual activity of 6%. Genetic analysis of *PFKM* gene demonstrated the presence of the heterozygote mutation c.1817 A>C (p.Asp543Ala) (6).

No family history of neurological or neuromuscular disorders was reported. His mother, sister, brother, and daughter had normal neurological examination as well as forearm test.

We re-evaluated this patient when he was 68-year-old. No significant clinical progression was reported until the age of about 54, when, after starting cyclosporine following liver transplantation for hepatocellular carcinoma, he started to progressively develop gait impairment up to either a need of walking aids at home or a wheelchair for longer distances.

Also of note in his previous clinical history, hypertension and, at least, two episodes of atrial fibrillation at 60 and 65 years of age were recorded leading to start amiodarone treatment.

**Figure 1:**
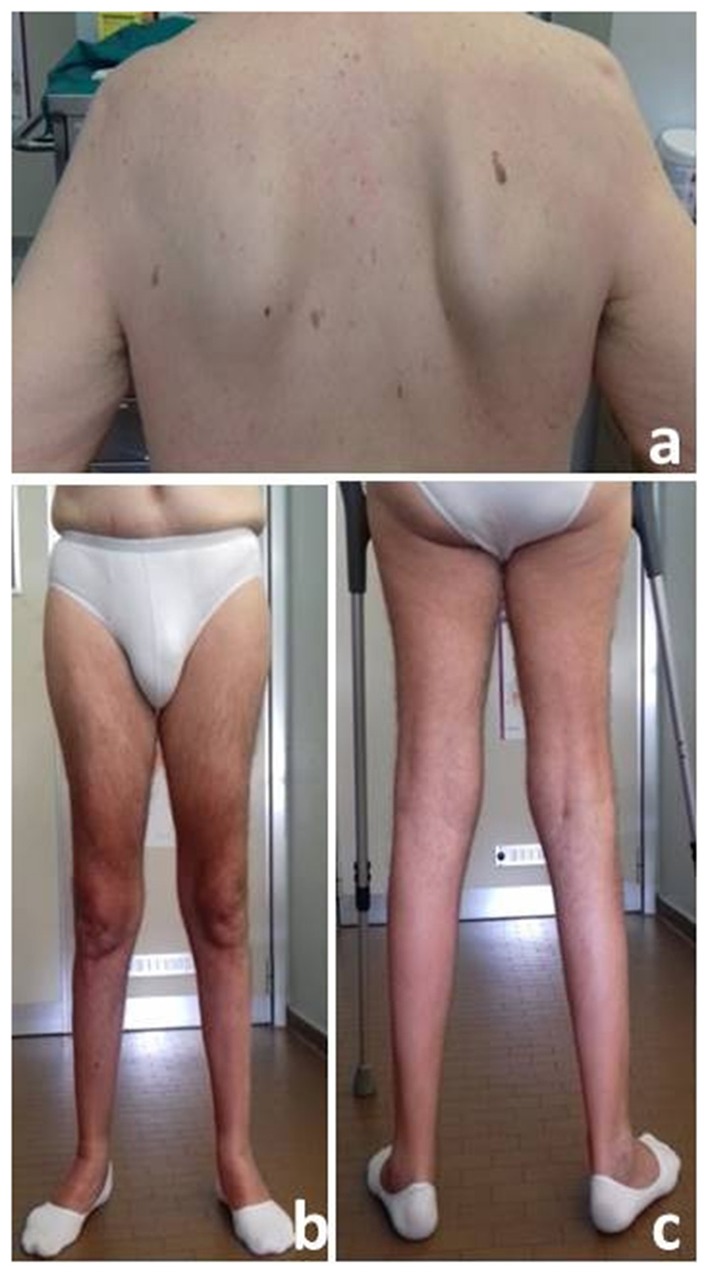
Winged Scapula **(a)** and moderate atrophy of anterior **(b)** and posterior **(c)** lower limb muscles.

Neurological examination revealed waddling and stepping gait, which was possible for short routes with both-sided support, inability to getting up from a sitting and squatting position, and positive Gowers' sign. Strength of the facial, bulbar, and neck muscles was normal. Symmetrical proximal and distal lower limb weakness evaluated by MRC scale (Medical Research Council Scale for Muscle Strength) was detected: thigh flexion 2/5; thigh extension 2/5; thigh adduction 4/5; thigh abduction 4/5; leg flexion and extension 4-/5; foot dorsal and plantar flexion 4-/5). Only a mild proximal weakness of upper limb (MRC 4/5) was observed. A moderate hypotrophy of quadriceps and leg muscles as well as winging scapula were detected (Figure 1). The ankle jerks and knee tendon reflexes were absent. No sensory abnormalities were observed except for a reduction of the sensory proprioception at the toes.

He was taking cyclosporine, mycophenolate, doxazosin, valsartan/hydrochlorothiazide, acetylsalicic acid, amiodarone, and allopurinol.

## Description of Laboratory Investigations and Diagnostic Tests

Endocrine, renal or liver dysfunctions, inflammatory or rheumatological disorders, electrolyte imbalance, vitamine deficiencies, hypoalbuminemia, and infections (hepatitis B and C, HIV, Borrelia Burgdorferi) were ruled out by appropriate laboratory investigations.

CK were highly increased, about 20-fold the normal values, in several occasions. Forearm test showed no rise in lactate with a normal rise in ammonia.

Electromyography (EMG) showed a diffuse myopathic pattern. No spontaneous activity was detected. Motor and sensory nerve conduction studies were unremarkable.

Genetic analysis of *PFKM* gene was conducted by direct sequencing on DNA extracted from peripheral blood. This analysis confirmed the heterozygote c.1817 A>C (p.Asp543Ala) mutation (6) and showed the presence of the heterozygote c.488 G>A (p.Arg100Gln) mutation on the second allele (4).

In the clinical follow-up, Holter ECG showed first-degree atrioventricular block with rare isolated and repetitive supraventricular ectopic beats and isolated ventricular ectopic beats. Trans-thoracic echocardiogram showed an aneurysm of the interatrial septum and a mild left ventricular hypertrophy. Pulmonary functional tests were normal.

Spine X rays documented a mild thoraco-lumbar scoliosis.

Brain and spinal cord MRI were normal.

**Figure 2:**
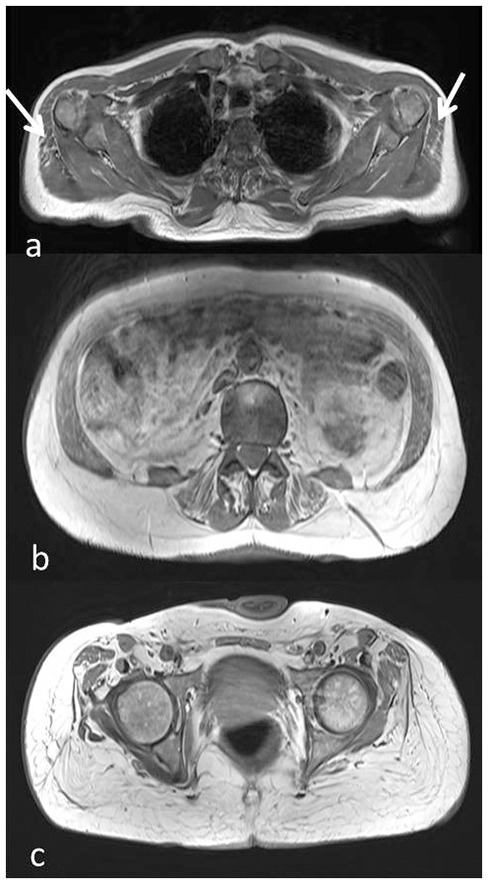
Muscle MRI axial T1-weighted images at the level of the shoulder girdle **(a)**, lumbar muscles **(b)**, and pelvic girdle **(c)**, showing minimal adipose involvement of the deltoid muscles **(a**, arrow), discrete fatty substitution at the level of the abdominal and lumbar muscles **(b)**, while gluteus maximus and medium are completely substituted **(c)**.

**Figure 3:**
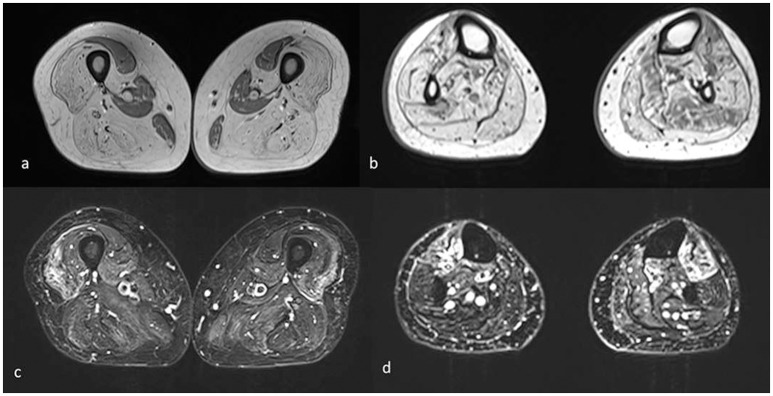
Muscle MRI axial T1 **(a,b)** and T2 STIR images **(c,d)** of the lower limbs shows mainly complete fatty substitution of both anterior and posterior thigh muscles, with selective sparing of the medial ones, specifically rectus, gracilis, sartorius, and adductor magnus **(a)**. Diffuse adipose involvement also of the leg muscles, less pronounced at the level of the soleus, and tibialis anterior muscles **(b)**. Inhomogeneous STIR hyperintensity was also associated, mainly in the quadriceps at the level of the thigh and in the tibialis anterior at the level of the leg **(c,d)**.

Muscle MRI showed adipose substitution of both anterior and posterior thigh muscles with selective sparing of the medial ones and, in particular, of rectus, sartorious, gracilis, and adductor longus muscles. Marked signs of adipose substitution were also documented in the legs with a selective replacement of gemelli and peroneus muscles and a relatively milder involvement of the soleus and tibialis anterior muscles. Shoulder girdle muscles showed minimal adipose involvement of the deltoid muscles, while face, abdominal, and lumbar muscles were spared (Figures 2, 3).

## Discussion

Among the four different forms of GSD VII deficiency, the classical presentation is the most common, being characterized by childhood-onset of myalgia and contractures following isometric or intense dynamic exercise, often associated with myoglobinuria (1, 3). Usually, the patients can live sufficiently well by adjusting their daily activity levels (1, 3). Jaundice reflecting hemolysis with high levels of bilirubin and hyperuricemia with gouty arthritis (due to the kidney damage resulting from processing myoglobin) have also been described (1, 3).

Infants with the severe and rapidly progressive infantile form have hypotonia at birth and develop severe fixed muscle weakness worsening over time, cardiomyopathy, respiratory failure, arthrogryposis, seizures, cortical blindness, corneal opacification. Death usually comes within 2 years (1, 3, 4).

In the late-onset form, a mild fixed myopathy with proximal muscle weakness and fatigue is the typical presentation. Many of the symptoms found in the classical type are absent in this form (1, 3). The weakness appears in adulthood, although some individuals have difficulty with sustained exercise starting in childhood (1, 3).

The hemolytic form is characterized by hemolytic anemia with no signs or symptoms of muscle involvement (1, 3, 4).

When initially evaluated, our patient presented with a clinical picture characterized by exercise intolerance, contractures, and myoglobinuria well-fitting with the classical form of PFK deficiency. Clinical features remained unchanged until late fifties when he developed a severely progressive muscle weakness, mainly involving the lower limbs, up to losing independent ambulation in <10 years.

The unexpectedly severe progression of the myopathy was confirmed by muscle MRI that showed muscle fatty replacement predominantly in the lower limbs that was not symmetrical. Deltoid, abdominal, and lumbar muscles were mildly involved while gluteus maximus and medium, both anterior and posterior thigh muscles and most of leg muscles were severely affected. On the other hand, a selective sparing of the rectus, sartorious, gracilis, and adductor longus muscles in the thighs as well as a relatively sparing of the soleus and tibialis anterior muscles in the legs was observed.

This is a quite unusual muscle MRI pattern. In fact, although there is a scarcity of muscle MRI studies in GSD VII, only very mild fatty infiltration in both soleus and peroneal muscles or no fatty degenerative changes have been observed in previous reports so far (7).

The unusual clinical and MRI findings of our patient might be related to a superimposed toxic myopathy from cyclosporine and amiodarone, since the clinical worsening started after the institution of these medications.

Indeed, it is well-known that cyclosporine may trigger neuropathy and myopathy as side effects (8, 9). The cyclosporine-induced myopathic damage usually presents as an acute condition following concomitant administration of statins or colchicine (8, 9). However, generalized myalgia and proximal muscle weakness, which develops within months after starting this medication, were also observed (5).

In a study including 34 patients who developed myopathy on cyclosporine, two of them received this drug on monotherapy, while, in the remaining cases, cyclosporine was administered with other myotoxic drugs such as statins or colchicine (10).

In muscle biopsies from patients affected by cyclosporine induced-myopathy, necrosis and non-specific type 2 muscle fiber atrophy were described, sometimes in association with signs of mitochondrial damage including ragged red fibers and lipid vacuoles (5). Pathogenic basis of the muscle damage is not completely understood and a destabilization of the lipophilic membrane leading to muscle fiber degeneration has been postulated (5).

Amiodarone is also known to exacerbate myotoxic effects of statins often causing (alone or in combination with statins) ataxia, neuropathy, and myopathy with severe rhabdomyolysis at onset (11–14). The amiodarone-related neuromyopathy is clinically characterized by severe proximal and distal weakness along with distal sensory loss and reduced muscle stretch reflexes (5). Muscle biopsy usually showed autophagic vacuoles and neurogenic atrophy (5). Amiodarone is believed to form drug-lipid complexes which are resistant to lysosomal enzyme digestion, thus resulting in the formation of autophagic vacuoles filled with myeloid debris (5).

In view of these considerations, in our patient, a relevant role for cyclosporine and amiodarone in inducing a prolonged toxic damage aggravating the pre-existing muscle disorder can be obviously considered.

Unfortunately, we were not able to repeat a muscle biopsy which could have given further valuable informations because the patient refused to repeat it. However, the temporal relationship between clinical worsening and starting cyclosporine as well as the chronic intake of amiodarone and the muscle MRI findings which were quite atypical for a GSD VII, strongly support the hypothesis that the patient clinical course cannot be solely related to Tarui's disease, but it is likely due to an additive chronic toxic damage caused by the two drugs.

To our knowledge, this is the first study reporting a severe muscle damage caused by concomitant administration of amiodarone and cyclosporine in a subject affected with a pre-existent myopathy.

Therefore, this case highlights that physicians should be aware of the administration of potentially myotoxic drugs in patients with myopathies, particularly when in association, as in the case of amiodarone and cyclosporine in our patient. Muscle MRI is very useful in defining the muscle damage distribution and may help in differential diagnosis and in identifying possible overlapping iatrogenic conditions.

## Ethics Statement

Diagnostic procedures were conducted in a routine diagnostic setting and therefore EC approvation was not necessary. Subject gave informed consent in accordance with the Declaration of Helsinki.

## Author Contributions

MF: Study conception an design, acquisition of data, analysis and interpretation of data, and drafting of manuscript. SCP: Acquisition of data, analysis and interpretation of data, and drafting of manuscript. AP: Acquisition of data, analysis and interpretation of data, and critical revision of manuscript. OM: Drafting and critical revision of manuscript. AG, FC, SGC, and EB: Acquisition of data and drafting of manuscript. RV: Acquisition of data, analysis and interpretation of data. AlP: Critical revision. AT: Analysis and interpretation of data, drafting of manuscript and critical revision.

### Conflict of Interest Statement

The authors declare that the research was conducted in the absence of any commercial or financial relationships that could be construed as a potential conflict of interest.
